# A Magnetically Tunable Check Valve Applied to a Lab-on-Chip Nitrite Sensor

**DOI:** 10.3390/s19214619

**Published:** 2019-10-24

**Authors:** Sean C. Morgan, Andre D. Hendricks, Mae L. Seto, Vincent J. Sieben

**Affiliations:** Department of Electrical and Computer Engineering, Dalhousie University, 1360 Barrington Street, Halifax, NS B3H 4R2, Canada; sean.morgan@dal.ca (S.C.M.); an639036@dal.ca (A.D.H.); mz715250@cs.dal.ca (M.L.S.)

**Keywords:** microfluidics, nanomolar nitrite, in situ marine sensor, lab-on-chip, valve

## Abstract

Presented here is the fabrication and characterization of a tunable microfluidic check valve for use in marine nutrient sensing. The ball-style valve makes use of a rare-earth permanent magnet, which exerts a pulling force to ensure it remains passively sealed until the prescribed cracking pressure is met. By adjusting the position of the magnet, the cracking pressure is shown to be customizable to meet design requirements. Further applicability is shown by integrating the valve into a poly(methyl methacrylate) (PMMA) lab-on-chip device with an integrated optical absorbance cell for nitrite detection in seawater. Micro-milling is used to manufacture both the valve and the micro-channel structures. The valve is characterized up to a flow rate of 14 mL min^−1^ and exhibits low leakage rates at high back pressures (<2 µL min^−1^ at ~350 kPa). It is low cost, requires no power, and is easily implemented on microfluidic platforms.

## 1. Introduction

Microfluidic devices have obtained global relevance since their initial application in miniaturized separation and chromatographic instrumentation [1]. More recently, micro-total-analysis systems (µTAS), have been applied to chemical analysis [2] and environmental monitoring [3,4]. The small, low-cost, power efficient nature of microfluidic analysis platforms have even been used for in situ ocean nutrient sensors in recent years [5,6]. Their potential to do automated and low-volume measurements at high pressures [7,8] makes them amenable for sensor deployment in deep ocean environments. However, reliable fluid handling remains a core development area in establishing higher levels of integration on microfluidic devices. This is typically accomplished with specialized micro-valves and micro-pumps [9,10].

Microfabricated valves fall into one of two categories: Active or passive. Active valves require external actuation and often rely on off-chip support systems. A typical active membrane valve uses induced fluid pressure to actuate a movable diaphragm to seal a channel [11]. Generating and controlling pressure for these on-chip active valves require a substantial amount of energy and external equipment that limit the ability to miniaturize. Complex in situ lab-on-chip systems employ multiple pneumatic or hydraulic actuators driven by off-chip solenoids. In the works by Ogilvie et al. [12] and Beaton et al. [13], completely automated lab-on-chip nutrient sensors contained 10–15 actuators. The solenoids contributed to a significant power draw during operation for fluid routing and handling, over 10 Watts, equating to 100 s if not 1000 s of Joules of energy per sample measurement. Using this much energy per sample limits the number of measurements possible per deployment with current battery capacities on small autonomous underwater vehicles and drifting floats. Incorporating rugged passive valves can reduce the energy demand for fluid handling on microfluidic devices.

Passive valves require no external power or interface and most often use simple mechanical biases, like an elastomeric membrane [14], or some other physical property to restrict fluid flow in one direction. The Tesla valve, for example, uses an in-channel geometric pattern to increase fluidic resistance in one direction and not the other [15]. Passive valves can also be made by exploiting capillary pressure differences in microchannels [16], or by using an air bladder to create a capillary stop-valve [17]. Other types of valves include using comb-like structures [18] or centrifugal elastic valves [19]. Flap valves are perhaps the most common and use a thin membrane that seals against a rigid body in one direction, and have freedom to deflect in the other [20]. Although flap valves generally seal well against reverse flow, they are still subject to leakage at low flow rates and do not have a strong seal in an unbiased state. Furthermore, such membrane valves are usually made up of at least two distinct materials, requiring unique bonding procedures between layers. This introduces extra manufacturing equipment and methods, such as UV exposure/oxygen plasma/chemical surface modifications and are prone to sealing failures if stringent quality controls are not implemented. Alternatively, a few researchers have implemented ball and cage valves [21] based on the Starr–Edwards valve used in heart surgery [22]. When forward flow is applied, the ball is dislodged from a seat and retained by the cage. In reverse flow, the ball is pushed into the seat, creating a seal. However, like flap valves, ball valves show appreciable leakage, on the order of 100 µL min^−1^ when a reverse pressure of 25 kPa is applied [23]. Backflow is unacceptable and must me be minimized on any in situ marine sensor, as reagent cross-contamination reduces the integrity and accuracy of the measurements.

Many microfluidic applications use magnetic fields to implement on-chip actuation without direct physical coupling. Micro-magnetofluids, for example, can be manipulated with an external magnetic field to perform on-chip mixing, pumping, or even droplet formation [24]. Yamahata et al. and Shen et al. [21,23] have explored the use of permanent magnets encased in a deformable membrane to act as a piston for a solenoid-based micropump. The reported systems use oscillating magnetic fields to drive an on-chip piston with two ball-style check valves to pump fluid. Other groups in the biomedical field have researched magnetic hydrogels [25,26,27] to create magnetically actuated microvalves [28,29]. These systems, however, are intended for use in benchtop applications and are not likely to withstand extreme pressure environments. Furthermore, the valves require more than 3 s to fully seal, which exceeds the activation time requirement for many applications. Single use drug delivery systems [30] have used permanent magnets as external valve actuators but are targeted for limited and disposable applications. Magnetorheological fluids can be actuated using a permanent magnet to seal a microchannel [31]. These innovative valves, however, are made with an intermediary PDMS layer, and partially seal 80–90% of the channel at extremely low flow rates (<0.1 µL min^−1^). Other common valve designs use magnetized polymers to create magnetically actuated membrane valves [32,33,34,35]. Valves of this sort, however, require the fabrication and bonding of specialized polymers. Many of the reported magnetic valves in the literature, as listed above, are activated with external linear translation stages or with energy intensive electronic systems. Passively biased magnetic valves are much less common but have potential for use in power restricted applications.

Here, we report the design of a tunable poly (methyl methacrylate) (PMMA) ball-type check valve. An embedded neodymium–iron–boron (NdFeB) permanent magnet was used to passively seal the valve with a tunable cracking pressure. The valves were created with a robust bonding procedure that simplifies the manufacture and integration of the valves with lab-on-chip systems. The valves exhibited very low leakage rates at high back pressures and an average cracking pressure of 18 ± 2 kPa in their default state. To demonstrate the utility of this valve design, we integrated it into a complete lab-on-chip nitrite sensor. The lab-on-chip nutrient sensor was made as a portable alternative to traditional instruments such as autoanalyzers, which are far too large and expensive for in-situ deployment on most autonomous surface or underwater platforms. Nitrite was detected with the standard colorimetric Griess approach based on optical absorbance spectroscopy, along with accompanying electronics and software to enable complete automation. The final sensor was calibrated using solutions of known standard concentrations, integrated on an unmanned surface vehicle, and shown to perform nitrite measurements when deployed in tank trials as described below.

## 2. Materials and Methods

### 2.1. Chemicals and Preparation

All chemicals and reagents were supplied by Fisher Chemical (Springfield Township, NJ, USA), unless otherwise stated. The nitrite standards for the sensor calibration were prepared via a serial dilution of a 1000 µM stock, made from 69 mg of sodium nitrite (NaNO_2_, CAS 7632-00-0, EMD Millipore, Darmstadt, Germany) diluted with 1 L of Milli-Q water. The Griess reagent for the colorimetric measurement was prepared in 500 mL portions by mixing 0.5 g of sulfanilamide (C_6_H_8_N_2_O_2_S, CAS 63-74-1), 5 mL of concentrated hydrochloric acid (HCl, A144-500), and 0.05 g of NEDD (N-(1-Naphthyl) ethylenediamine dihydrochloride) (C_12_H_14_N_2_2HCl, 42399-0250) before dilution to volume with Milli-Q water. The chip bonding procedure required both chloroform (CHCl_3_, C607-4) and isopropyl alcohol (C_3_H_8_O, A451-4).

### 2.2. Valve Design

**Figure 1:**
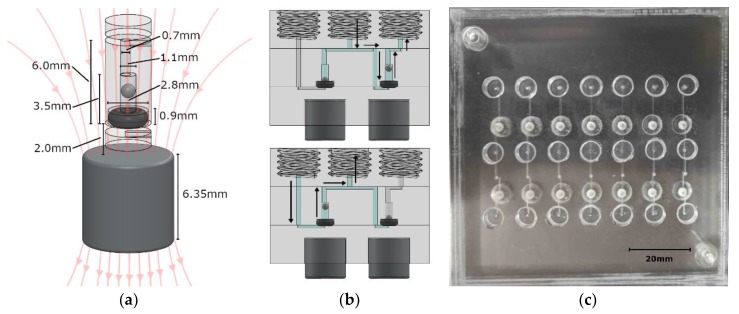
(**a**) CAD view of a single check valve, where the magnet position is adjusted/set to control the cracking pressure. (**b**) A valve system that is based on two check valves. The top image shows fluid dispensing, while the bottom image shows fluid withdrawal. A syringe screws into the center thread, while fluid lines connect to the inlet on the left and outlet on the right. (**c**) Photograph of a panel of valve systems used for testing and characterization.

Figure 1a is a three-dimensional (3D) rendering of the valve design, along with dimensions. The chip itself, as well as all the features (including the valves), was cut out of 12 × 12-inch PMMA sheets that were a quarter-inch thick (8505K734, McMaster-Carr, Elmhurst, IL, USA) using an LPKF S103 micro-mill (LPKF, Garbsen, Germany). PMMA was selected as our demonstration material because when bonded to itself, it is capable of withstanding high pressures without delaminating; please refer to previous work in ocean deployments [36,37].

In literature, microfluidic devices fabricated from solvent-bonded layers of PMMA have been shown to withstand high enough pressures for ocean deployments; in some cases, the chips survive ambient pressures >30 MPa [38]. Sun et al. [8] showed that chloroform (CHCl_3_) is an excellent choice for solvent-bonding PMMA as the two substances have a similar Hildebrand solubility parameter. They quantified the shear strength of the solvent bond between the PMMA sheets to be >3 MPa. Ogilvie et al. [39] used a similar method of solvent bonding using chloroform, and quantified the peak peel force of two bonded substrates to be >3.5 N/mm. The chloroform vapor exposure also decreased the surface roughness of the substrates, while still preserving the integrity of the microfabricated structures. The same bonding method has been used to fabricate devices deployed to depths of >140 m with no issues [36,37].

The valve was constructed with a 001 soft Viton O-ring (1284N101, McMaster-Carr, Elmhurst, IL, USA), a stainless steel 440C ball bearing (1598K16, McMaster-Carr, Elmhurst, Illinois, USA), an N-40 rare-earth (NdFeB) permanent magnet (NSN0617, MagCraft, Vienna, VA, USA), and three PMMA discs with microfabricated features. The stainless steel ball was 1 mm in diameter, while the cylindrical recess in which it was housed was milled to be 1.1 mm in diameter and 2.4 mm high, allowing liquid to flow around it when the valve was open. Above the ball recess was a 0.7 mm diameter via to the top of the center layer, where it met a fluid channel. The via was offset by 0.15 mm to the cylindrical axis of the valve to ensure that the ball did not act as a plug when brought to the top of the recess. Beneath the ball sat the 001 sized O-ring. The slot into which it was seated was 0.9 mm deep and 2.8 mm in diameter. The O-ring was pressed between the middle and bottom layers of PMMA to hold it in place. A fluid channel in the bottom PMMA layer passed beneath the O-ring to meet the valve chamber in the central layer and form a fluidic connection. When fluid is applied against the allowable flow direction of the valve, the steel ball will be pressed into the O-ring to create a seal.

Beneath the valve, embedded in the PMMA, was the N-40 grade cylindrical NdFeB permanent magnet (displayed in Figure 1b). It was 6.35 mm in diameter and 6.35 mm in height, exerting a calculated force of 0.0152 N on the ball, resulting in a theoretical cracking pressure of 19.41 kPa. This served to keep the valve closed until the cracking pressure was overcome, which prevented backflow during periods of inactivity. Both finite element method (FEM) simulations and analytical calculations were used to determine the force on the steel ball. Approximating the ball to be a single magnetic dipole in comparison to the permanent magnet resulted in a force calculation of [40,41]:

(1) $$F\left(z\right)≃-m\left(z\right)\frac{B_{r}R^{2}}{2}\left(\frac{{\left({\left(z+D\right)}^{2}+R^{2}\right)}^{3/2}-{\left(z^{2}+R^{2}\right)}^{3/2}}{{\left(z^{2}+R^{2}\right)}^{3/2}{\left({\left(z+D\right)}^{2}+R^{2}\right)}^{3/2}}\right)$$

where *R* and *D* were the radius and height of the cylindrical magnet, *B_r_* was the remanence field of the permanent magnet, and *m*(*z*) was the magnetic moment of the ball at height *z* above the surface of the magnet.

Figure 1b is a computer rendering of a simple two-valve system for withdrawing fluid from one source (inlet) and dispensing to another (outlet). A syringe is screwed into the center ¼-28 threaded port, while fluid lines are screwed into the outer two threaded ports. The top image is the valve system state while the syringe is dispensing, and the bottom image is the valve system state during withdrawal. Figure 1c is a testing panel for seven of these valve systems. This basic set up was used for characterization of our magnetically tunable valves. Each valve footprint in the plane of the chip was a circle that was 2.8 mm in diameter and 6.15 mm^2^ in area. The height of each valve was 3.5 mm. The lab-on-chip devices described here were 18 mm thick due to the three 6.35 mm PMMA sheets used, but thinner sheets can be used. The thicker PMMA sheets allowed for integral threaded access ports. The actual fluid volume inside the valves was <3 µL per valve, which is less than the volumes of typical microchannel structures used in lab-on-chip sensors. For example, a microfluidic serpentine mixer that is 200 µm wide by 200 µm deep and 250 mm long would have a total volume of 10 µL. By integrating our valve within a lab-on-chip system, we have removed interconnect volumes that are often far greater than the total volume on-chip. Two separate cylindrical magnets were used in the testing system that ensured an axially symmetric magnetic field through the steel ball. The external permanent magnet was 6.35 mm diameter and height in this case; however, a bar magnet could be used for dozens of valves on a single chip.

### 2.3. Valve Characterization Method

The valves were characterized when flow with Milli-Q water was applied in forward and reverse directions. A Honeywell in-line differential pressure gauge (26PCFFA6G, Digi-Key, Thief River Falls, MN, USA) was used for all pressure measurements, and a National Instruments USB-6009 DAQ (National Instruments, Austin, TX, USA) and Lab-View were used for data acquisition. The reported range of the pressure sensor was ±100 mV, which corresponded to ±100 psi. The resolution and offset of the gauge were measured by taking the root mean square (RMS) of a blank reading at 1000 Hz. All subsequent measurements were taken at a sampling rate of 1000 Hz, with the measured pressure offset considered. Furthermore, the range on the DAQ was set to ±500 mV, which meant the bit resolution of the analog to digital conversion was well within the error of the pressure sensor noise. The pressure sensor was fluidically connected to the chip through a T-junction (IDEX, Lake Forest, IL, USA) at either the inlet or outlet of the valve system. The pressure drop through the tubing was measured separately and subtracted from all measurements reported.

Backflow leakage rates were calculated using outlet pressure measurements. A bench top KD Scientific syringe pump (788101, Legato 101, KD Scientific, Holliston, MA, USA) was used to inject discrete volumes of water backwards into the valve in a step-like fashion. The subsequent increases in pressure were recorded and used to determine a pressure-to-volume ratio, which was then used to calculate the back-leakage rate over a 20-min window. The data were collected as 1 s averages of the signal sampled at 1000 Hz. This process was repeated for 5 different set points, where volumes from 10 to 80 µL were pumped into the valve outlet to achieve a dead-end pressure test. This equated to back pressures of approximately 40 to 380 kPa, respectively.

Forward flow characterization was done by using the syringe pumps to inject Milli-Q water into the valve inlet at set rates. The syringe pump was programmed to inject for 10 s at each flow rate. The measured pressure at these set flow rates was averaged and the standard deviation was recorded. Raw measurements were taken at a 1000 Hz sampling rate and the data reported are from a 0.1 s moving average window.

To determine the cracking pressure of the valves experimentally, the syringe pump was set to inject fluid into the valve inlet at a rate of 0.05 mL min^−1^. The measured pressure peaked at the cracking point and then dropped off once the valve was opened and fluid was moving through. The process was repeated with five different magnetic field strengths to show the tunability of the cracking pressure. This was accomplished by using a permanent magnet positioned at increasing distances from the ball seat in the check valve.

### 2.4. Microfluidic Chip Fabrication

**Figure 2:**
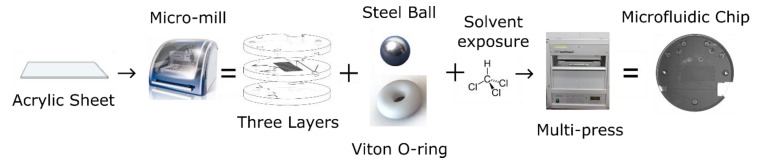
Process steps for manufacturing microfluidic chips with integral check valves.

**Figure 3:**
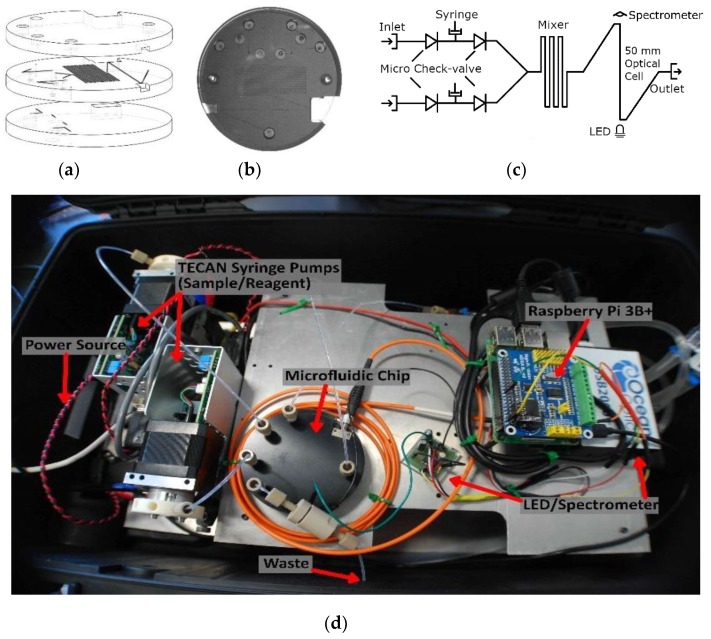
(**a**) Microfluidic chip design; (**b**) photograph of the microfluidic chip with integrated valves; (**c**) flow schematic; (**d**) lab-on-chip sensor housed in a water-tight case with labeled components.

The valves were integrated into a three-layer, tinted PMMA microfluidic chip made for use in the nitrite sensor. Figure 2 shows the flow of events involved in the fabrication of the microfluidic chip. The features of each chip layer were first milled into a 6.35-mm-thick sheet of tinted PMMA using the micro-mill. Both the channels and the absorbance cell were cut to have a square cross section with a height and width of 400 µm, and all vias were drilled to have a 0.7 mm diameter. Figure 3a shows the three individual layers of the chip before they are bonded. The bottom layer of the chip mainly served to seal the base of the valves and provide a housing for the permanent magnets, ensuring they were axially aligned to the valves. The central layer contained most of the features and channels. The recesses for the valve parts (O-rings and ball bearings) were milled into the bottom side of the central layer. The optical cell and mixing channels were milled into the top side of the central layer. The top layer provided external fluidic connections via threaded ports to external tubing and also provided a cap to seal the channels of the central layer. Receptacles for the LED and optical fiber were milled between the top and central layers; their dimensions and positions were designed to ensure optical alignment.

After the three discs were cut out, they were cleaned and their surfaces prepared for bonding. Following the steps provided by Ogilvie et al. for PMMA bonding [42], the discs were scrubbed under hot water with a brush and dish soap, rinsed with both tap water and Milli-Q, dried with pressurized air, rinsed with isopropyl alcohol, and then dried again. The O-rings and ball bearings were then set into their respective grooves in the central layer. Next, petri dish bases were placed on a hot plate set to 30 °C and filled with chloroform up to a pre-marked point. The marked point ensured the liquid level was ~2 mm from the surface of each disc. Permanent magnets were then used to suspend each PMMA disc from the lids of the petri dishes, with the bonding side facing down. The central layer had to bond on both sides, but it was decided that only the top side would face down, to prevent the O-rings and ball bearings from falling out. The petri dish lids with the attached PMMA discs were then set onto the petri dish bases filled with chloroform. Substrates were exposed for 5 min and then removed from the vapor. The discs were manually aligned and pushed together to form a preliminary bond. The three-layer puck was then inserted into an LPKF PCB Multipress II (LPKF, Garbsen, Germany) and pressed together with 6.25 MPa of pressure at 85 °C for 2 h to ensure a solid bond. Figure 3b shows a photograph of a microfluidic chip with 4 integral check valves, bonded in this manner.

Figure 3c is a fluid schematic of the nitrite chip design. Two Tecan syringe pumps (733085-B: Cavro XCalibur, Tecan Trading AG, Männedorf, Switzerland) were used to withdraw and inject fluid. The pumps were controlled by a Raspberry Pi 3B+ via a USB-serial connection. The fluids were mixed in a 0.75-m-long serpentine microchannel, and then analyzed in the on-chip optical absorbance cell before reaching the outlet. The integral absorbance cell was manufactured in tinted PMMA to prevent light scattering within the chip and to reduce the influence of background light [5]. The light source was a 525 nm LED (C503B-GAN, Cree Inc., Durham, CA, USA) that was optically coupled to the absorbance cell through a 1-mm-thick window of tinted plastic. The light output from the absorbance cell was coupled into an SMA fiber optic patch cable (M92L02, Thor Labs Inc., Newton, NJ, USA) to the detector, which was an Ocean Optics spectrometer (USB 2000+, Ocean Optics, Largo, FL, USA). A custom scripting language was created to control and coordinate the pumps, the spectrometer, and the LED driver. The components were mounted to a custom aluminum frame and enclosed in a water-tight Pelican case, as shown in Figure 3d, for deployment on an unmanned surface vehicle. There are smaller spectrometers commercially available like the STS miniature units from Ocean Optics. However, the sensor platform described above was primarily created as a rapidly re-configurable testbed. The platform will be used to verify new microfluidic chip designs and concepts for a variety of chemistries. Since the majority of colorimetric assays are based on single or dual wavelength absorbance measurements, the final lab-on-chip sensors do not need to use a spectrometer. A more compact lab-on-chip sensor can be realized using a photodiode in place of the spectrometer, as demonstrated by both Sieben et al. and Nightingale et al. [5,6].

## 3. Results and Discussion

**Figure 4:**
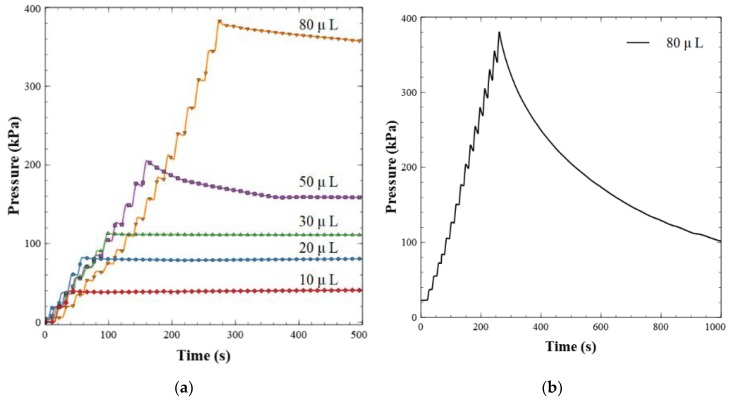
(**a**) Dead-end pressure test for leakage characterization. Fluid is injected backwards into the valve in 5 µL increments and then held after a total volume has been reached as shown. (**b**) Example of a failed valve. The pressure did not hold or stabilize over the 20-min window when fluid was injected backwards into the valve.

Figure 4a shows the results of the dead-end pressure test described above. Here, 5 µL increments of Milli-Q water were delivered to the valve outlet until a volume setpoint was reached, ranging from 10 to 80 µL. This can be observed by the staircase climb to the peak pressure value for each run. At pressures below ~150 kPa, the backflow was insignificant, less than <0.4 µL min^−1^. The stable pressure plateau observed from these dead-end pressure tests indicates excellent valve sealing—red, blue, and green data sets. At higher back pressures the valves did exhibit small leakage rates, less than 2 µL min^−1^. The leakage behavior at pressures above ~150 kPa showed a greater degree of variation with a standard deviation of 0.65 µL min^−1^, indicating that some valves leaked more than others. The maximum pressure expected from the final nitrite sensor in this and future work is 100 kPa, based on flow rates and channel geometries. Therefore, these valves are more than adequate and will not have leakage issues. Other ball-style check valves of similar designs [21,43] have leakage rates that are orders of magnitude higher than the work presented here; up to ~1 mL min^−1^ at 40 kPa. Furthermore, Oh and Ahn [44] surveyed 22 passive valves and only one had a lower leakage rate than our valve design. The valve was a membrane-based hexagonal flap valve made from ion etching and photolithography, with a leakage rate of 0.01 µL min^−1^ at 600 kPa. Other reported flap valves [45,46,47] have leakage rates exceeding 30 µL min^−1^. The leakage rate of our magnetically tunable valve is comparable or significantly lower than reported by others, while not relying on a cleanroom for fabrication.

During the manufacturing process, we observed a final yield of 75%. When the valves would not function correctly, the most frequent issue was an ineffective sealing against backpressure. Figure 4b is the back pressure drop of a valve with a non-functional seal; the majority of the fluid leaked across the valve over the 20-min window. This was most likely due to imperfect alignment during the bonding of the PMMA sheets, as visually observed. Misalignment would lead to O-ring deformation or improper seating in the PMMA groove during bonding. We noticed that the misalignment occurred primarily when the substrates/PMMA sheets were pressed together in the hydraulic press. This could have happened as the pressure distribution of the Multipress II is not guaranteed to be uniform, particularly if the chip was not centered perfectly. The alignment issue was partially mitigated by embedding at least three steel dowels in the chip before it was pressed. Alternatively, fit-for-purpose jigs to hold the chips in the hydraulic press can be created to improve bonding alignment.

**Figure 5:**
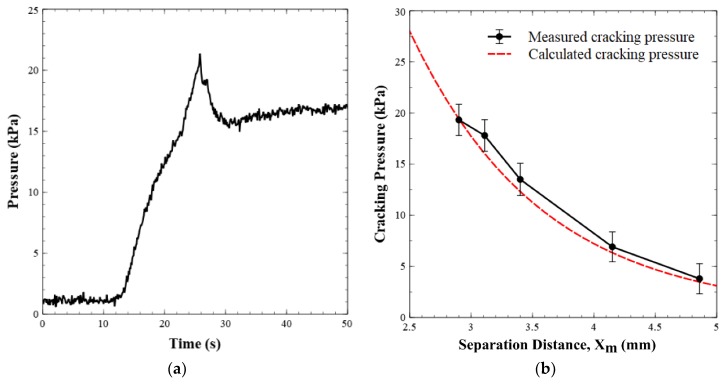
(**a**) Pressure profile of a valve as it opens under forward flow conditions. Milli-Q water is injected into the valve inlet at 0.05 mL min^−1^. Pressure increases until the point where it overcomes the magnetic pulling force on the steel ball. After the valve opens, flow occurs and pressure drops. (**b**) Experimental and calculated cracking pressures of the valve versus separation distance between the permanent magnet and the valve seat.

Figure 5a displays the pressure versus time when the valve is operated in the forward flow mode. This demonstrates that the pressure in the valve increased to the cracking point, before it dropped to a steady state pressure of ~17 kPa for a flow rate of 0.05 mL min^−1^. Figure 5b shows the cracking pressure versus the separation distance between the permanent magnet and the valve seat. The graph shows two data sets, one for the calculated/expected cracking pressure (given the separation distance) and one for the experimentally observed cracking pressure (*n* = 6 for each data point). Equation (1) was used to generate the theoretical curve of the cracking pressure as the separation distance was increased. Analytically, the cracking pressure at the default magnet position (2.9 mm separation distance) was calculated to be 19.41 kPa and it was experimentally found to be 18 ± 2 kPa, or within experimental error. The minor difference between the two values was likely due to uncertainty in the exact position of the ball, as there will be slight deformation of the O-ring as the ball compresses the rubber/Viton. Within the ranges tested, our experimental cracking pressures agreed with the calculated or predicted values.

**Figure 6:**
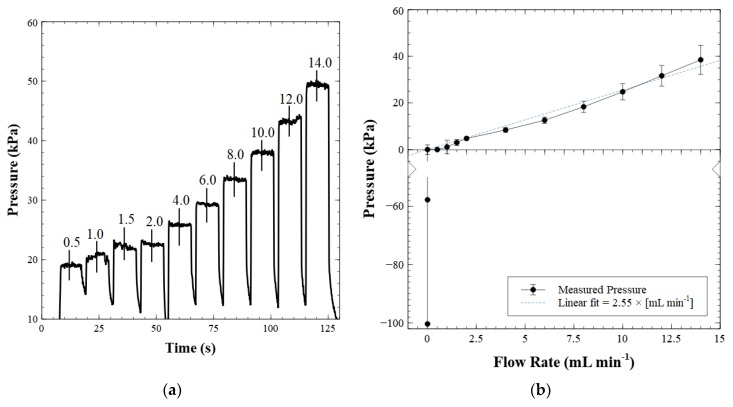
(**a**) Forward flow characterization. Fluid is injected into the valve inlet at set flow rates as labeled (mL min^−1^) in 10 s intervals. The pressures corresponding to each flow rate are measured as the average of each plateau, with the standard deviation about the average used as the experimental error; (**b**) characterization curve of the valve. The cracking pressure of the valve is subtracted from each measurement. The displayed error is the standard deviation from the results of six different test valves.

Figure 6a shows pressure versus time for a series of flow rates applied to the valve inlet ranging from 0.5 to 14 mL min^−1^. Figure 6b is a characterization curve constructed from both the forward pressure and back pressure measurements, where the cracking pressure of the valve is subtracted from each measurement. At flow rates greater than 1 mL min^−1^, the pressure increase was proportional to the increase in volumetric flow rate, as expected. However, we noticed that the pressure measured at flow rates less than 1 mL min^−1^ did not fit the linear trend. We attribute this behavior to the force experienced by the steel ball in the recess after the valve cracks open. At low flow rates, post-cracking, the magnetic force on the ball is at its relative maximum. As the ball moved further into the recess, the pull from the magnet decreases quadratically with distance, reducing the required pressure to keep the ball off the valve seat. This opposition of forces between fluid pressure and magnetic pull continued until the ball reached the top of the recess where it could move no further. After this point, the change in flow rate was the only factor affecting the pressure measurements, causing the pressure to increase linearly with flow rate. The error in this chart was calculated as the standard deviation of the averaged results of six different test valves. The tunable nature of these check valves enables them to be integrated with a variety of micro-pumps. For example, piezoelectric actuators are often used in conjunction with two valves [48], much like our valve system in Figure 3. Using our valves would allow slight adjustments to the cracking pressures post-manufacture by altering the magnet separation/placement, thereby enabling the valves to function optimally during pumping. The possibility of magnetically tuning the cracking pressure of a passive valve has, to the authors’ knowledge, not been explored elsewhere. Further, by not incorporating a mechanical spring in the check valve design, we greatly simplify the fabrication process and extend service life.

### Application: Nitrite Sensor

**Figure 7:**
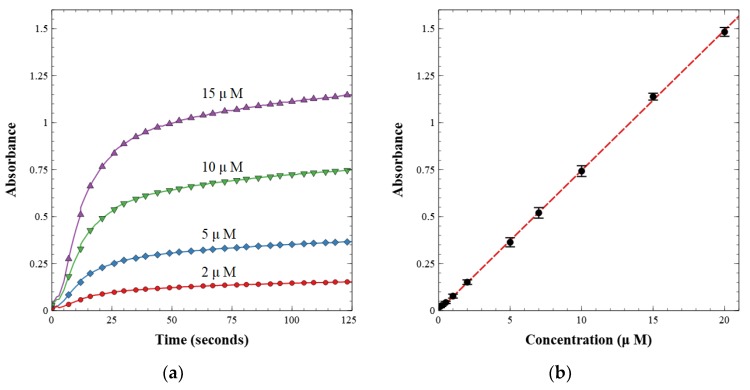
(**a**) Absorbance over time of pre-made standard nitrite concentrations; (**b**) calibration curve of nitrite lab-on-chip sensor showing absorbance versus concentration.

The working valves were then integrated into a complete nitrite sensor. The sensor was calibrated with known nitrite concentrations between 0.1 and 20.0 µM. First, a light reference measurement was acquired with the LED on and a blank water sample in the cell. Second, a dark reference measurement was acquired with the LED off. The light and dark spectra were stored and used to calculate absorbance from the intensity measured during subsequent sample readings. The prepared nitrite solutions were then injected one at a time and mixed with the reagent at a 1:1 volumetric ratio. Each fluid was injected at a rate of 250 µL min^−1^ for a total of 500 µL min^−1^. Flow was then stopped, and the solution was held in the optical cell for 125 s to allow for color development. The last 5 s of each data set were averaged and used as the absorbance measurement for each concentration. A linear calibration curve was then built from these measurements as per the Beer–Lambert law, shown in Figure 7. Figure 7a displays the absorbance over time as the azo dye developed from the reagent and nitrite standard sample. Figure 7b is the resultant calibration curve, relating absorbance to concentration.

**Figure 8:**
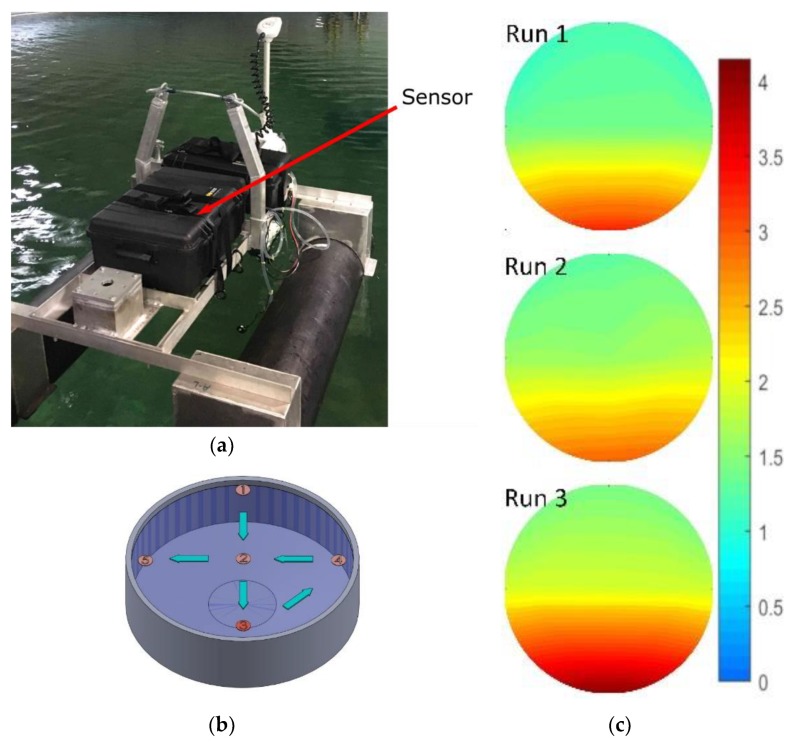
(**a**) Sensor in a water-tight case mounted to an unmanned surface vehicle in the Aquatron; (**b**) Aquatron CAD model with sampling points labeled. Nitrite dosing was done at point 3; (**c**) concentration mapping of three consecutive runs, scale units displayed are micromole.

Next, the sensor was mounted to an unmanned surface vehicle, as shown in Figure 8a. The combined platform and sensor were tested in a large tank of seawater at the Dalhousie University Aquatron facility. The Aquatron main tank was 15.24 m in diameter and held 684,050 L of water pumped directly from the Bedford Basin, Nova Scotia, Canada. The Basin had a nitrite concentration ranging from 0.1–0.6 µM at the time of study. The initial concentration of the tank was adjusted to 1 µM to ensure a uniform starting point.

The sensor and platform were then deployed three times to gather spatial concentration data, while we simultaneously delivered nitrite into the tank at a known point. For each of the three consecutive runs, we gradually added 1 L of 100 mM nitrite solution into the tank at sampling point three, shown in Figure 8b. Next, the vehicle with our sensor was driven around the tank, stopping at each of the waypoints shown in Figure 8b. At each waypoint, the concentration was measured as the average of five readings taken over a 5-min window. For the vehicle tests, we shortened the sensor color development time from 2 min as per the calibration above, down to 45 s, in an effort to speed up the sampling rate. The final 75 measurements, 25 from each run, are displayed in Figure 8c as an intensity map of concentration over the surface of the Aquatron. The Aquatron was equipped with recirculation pumps that effectively moved the high concentration of nitrite from the delivery point (6 o′clock) to the drainage point in the center of the tank. Furthermore, the background nitrite concentration gradually increased between runs as expected, given the amount of added nitrite. The background nitrite concentration increased from 1.0 to 1.2 µM between the initial level and run 1, from 1.2 to 1.3 µM between runs 1 and 2, and from 1.3 to 1.5 µM between runs 2 and 3. The advancing nitrite front and the overall increase in the background concentration were observed in all three runs, thereby successfully demonstrating our tunable check valves integrated with a fully automated lab-on-chip nitrite sensor.

## 4. Conclusions

The valve presented here was made for the purpose of marine sensing but has widespread applicability. Most microfluidic device requires fluid control mechanisms, and a passive, simple, tunable check valve enables lab-on-chip designs flexibility in adjusting cracking pressure post-creation. The valve was integrated into a microfluidic chip and used in a working nitrite sensor, both in the lab and in a seawater filled tank environment. Future pressure cycle tests and computer simulations will be performed to evaluate the robustness of the valves and their integrity at high pressures.
